# Global Collaborations in Developmental Dysplasia of the Hip

**DOI:** 10.1007/s43465-021-00504-4

**Published:** 2021-09-04

**Authors:** Kishore Mulpuri, Emily K. Schaeffer, Charles T. Price

**Affiliations:** 1 Department of Orthopaedics, University of British Columbia, Vancouver, BC Canada; 2 Department of Orthopaedic Surgery, British Columbia Children’s Hospital, 1D.69 – 4480 Oak Street, Vancouver, BC V6H 3N1 Canada; 3 University of Central Florida College of Medicine, Orlando, FL USA

Developmental dysplasia of the hip (DDH) is the most common pediatric hip disorder, and despite decades of research, there is very little high-level evidence to inform health care providers, patients and families on the diagnosis, treatment and management of this condition. There are evident knowledge gaps in DDH screening and management, which can have important and long-lasting effects on children and their families. The impact of screening and detection efforts is significant, both in the number of children and families affected, and in cost, time and personnel resources to medical systems. Early detection is critical to mitigate the potentially substantial long-term burden of disease. Even when treated, DDH is a major cause of early hip replacement or osteoarthritis in young adults.

A 2011 review by Loder and Skopelja on the epidemiology and demographics of hip dysplasia highlighted challenges posed by the complex nature of the DDH pathology spectrum [1]. They report a range in incidence per 1000 live births from 0.06 in Africans to 76.1 in Native Americans. Additionally, the authors identify significant variability in incidence within racial groups across geographic locations [1]. These findings emphasize the importance to recognize that studies on DDH in specific regions cannot be generalized to other parts of the world. It is imperative to take a globally equitable, diverse and inclusive approach to understand the full scope of the impact of DDH and identify best practices to optimize outcomes for children worldwide.

Screening practices for DDH vary widely across centres and between regions, and may depend on local resource availability. Multi-centre, prospective studies and collaborations have the potential to generate high-quality evidence to guide screening and management practices, but typically fail to include a truly global representation of the patient population. There can be critical differences in presenting patient populations in different regions that will provide better insight into how best to optimize outcomes for DDH. Much of the existing research has been performed in and holds relevance to the Global North. In contrast, the Global South has been largely overlooked in multi-centre collaborative efforts despite comprising significantly more of the world’s population. To advance DDH care and outcomes for children and families around the world, multi-centre studies and collaborations must truly reflect the global population.

We established the International Hip Dysplasia Registry (IHDR) with the principles of global equity, diversity and inclusion (EDI) at the forefront. IHDR evolved from a smaller study within the auspices of the International Hip Dysplasia Institute (IHDI), consisting of eight centres in North America, Australia and the United Kingdom (UK). This study examined early outcomes of patients diagnosed with a dislocated hip at rest under 18 months of age, and findings demonstrated distinct differences in presenting patient population demographics and DDH severity across contributing centres [2]. We also found that while swaddling was associated with late-presenting cases, typical DDH risk factors were less prevalent in this group [3]. Additional findings around bracing, closed and open reduction demonstrated good outcomes [4, 5], but the study in general served to highlight outstanding questions about optimal screening and management practices, even in developed countries [6]. Lack of global representation within the study prevented generalization of results, and served as the impetus to expand its scope and transition into IHDR in 2016.

The registry has since expanded to 29 centres across eight countries on five continents to become the largest prospective study on DDH in the world. We are also actively seeking to further include under-represented regions through strategic addition of centres in those areas (Fig. 1). Beyond North America, Australia and the UK, IHDR first expanded to include five centres in India. Without a dedicated screening programme or pathway in India, late-presenting or late-detected DDH is an issue often necessitating more invasive surgical approaches to treatment that can have long-term impact on a child’s mobility and quality of life. Indeed, we have seen within IHDR that the mean age at presentation is older amongst children from the contributing centres in India compared to other registry centres. Understanding these differences will be critical to identifying optimal screening methods and treatment strategies that can be applied while accounting for the regional context.

**Fig. 1:**
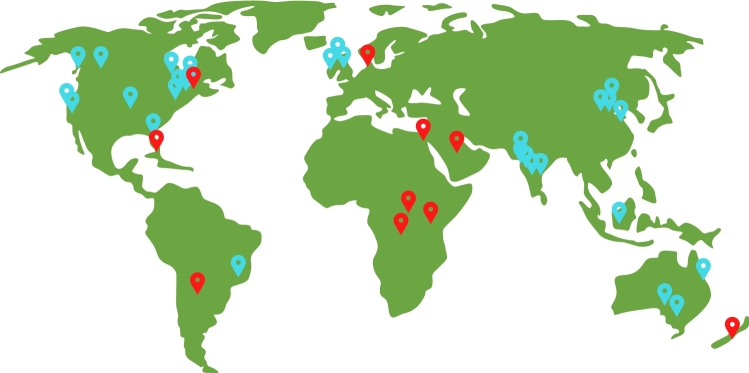
World map depicting existing collaborations (blue pins) and intended future collaborations (red pins) within the International Hip Dysplasia Registry (IHDR)

Recently, surpassing 4000 patients recruited worldwide, IHDR is ideally positioned to carry out targeted hypothesis-testing studies on long-standing areas of controversy or equipoise in the field. Specifically, we can design and execute randomized controlled trials on the necessity of brace treatment in radiological dysplasia, brace treatment for Barlow positive hips and the extent of follow-up required for children with DDH risk factors, as well as comparative effectiveness studies for rigid and dynamic braces. Historically, these studies have been limited to single-centre retrospective designs lacking adequate sample size and power to provide high-level evidence. IHDR possesses the size and scope to perform these trials with relevance in a global context.

The expansion of IHDR has also facilitated rich collaborations beyond the original scope of the research registry, including the opportunity for culturally specific care pathway development. For example, key members of IHDR acted as external facilitators and local champions for the development of an India-specific DDH care pathway. This process brought together experts from seven national organizations representing over 120,000 healthcare professionals practicing in India with the goal of standardizing a DDH screening process within the limits of local resources. This process can now be used as a model to develop similarly culturally specific care pathways in other regions and countries, adapting for local resource availabilities and practicalities. We have adopted a “pay it forward” approach to implementing this model in new regions, whereby a local champion involved in pathway development in their own country lends their expertise and advises local champions elsewhere.

Global collaborations also provide valuable opportunities for knowledge exchange and enhancement of clinical care, both formally though educational fellowships and visiting professor programmes, and informally through peer mentorship and case discussions. India and China have high operating room (OR) volumes for patients with DDH, due in part to the vast population and in part to the tendency for later age at presentation. Consequently, countries and regions, such as the United States, Canada, Europe and Australia, can learn critical lessons from surgeons in India and China who deal with these high OR volumes during their regular practice. Bidirectional visiting fellowships can facilitate this critical knowledge exchange and improve our collective understanding for which procedures can optimize long-term results for children.

The inherent value of equity, diversity and inclusion highlights the need to be a global citizen in research collaborations. We urge all centres and individual investigators involved in prospective, multi-centre studies to reflect on how truly globally representative the effort is, and take steps to diversify and include under-represented regions with rich patient populations. The need to be globally inclusive expands beyond just collaborating investigators, however. We must also consider patient and family partnerships to guide the development of research priorities and maximize impact through knowledge translation (KT). While we have much more work to do in this aspect, IHDR has partnered with seven DDH advocacy groups—IHDI, Healthy Hips Australia, I’m a HIPpy, Steps Charity Worldwide, DDH UK, Spica Life and Miles4Hips—to create the IHDR KT Advisory Board. In collaboration with these organizations, we recently conducted a survey study aimed at better understanding the caregiver experience throughout care for DDH [7]. Findings demonstrated caregivers experienced a significant burden due to DDH diagnosis and treatment, and that global education efforts to raise awareness and improve screening, diagnosis and monitoring of at-risk infants are needed [7]. Understanding the impact on and priorities of patients and families experiencing the DDH journey are vital to informing future research.

We recently celebrated the first annual International Hip Health Day (IHHD) on May 31, 2021. This virtual event brought together surgeons, researchers, study groups, patients and advocates to recognize the broad scope of work being done around the world in different areas of specialization (i.e. research, clinical care, education and advocacy). At the event, we honoured Hip Legends from each of these realms, recognizing their tireless and important contributions to advancing hip health globally. We need more initiatives such as this, both regionally and globally, to continue to promote collaboration and further improve the quality of life of children with DDH worldwide.

## References

[1] Loder RT, Skopelja EN (2011). The epidemiology and demographics of hip dysplasia. ISRN Orthopedics.

[2] Mulpuri K, Schaeffer EK, Kelley SP (2016). What is the impact of center variability in a multicenter international prospective observational study on developmental dysplasia of the hip?. Clinical Orthopaedics and Related Research.

[3] Mulpuri K, Schaeffer EK, Andrade J (2016). What risk factors and characteristics are associated with late-presenting dislocations of the hip in infants?. Clinical Orthopaedics and Related Research.

[4] Upasani VV, Bomar JD, Matheney TH (2016). Evaluation of brace treatment for infant hip dislocation in a prospective cohort: Defining the success rate and variables associated with failure. Journal of Bone and Joint Surgery. American Volume.

[5] Sankar WN, Gornitzky AL, Clarke NMP (2019). Closed reduction for developmental dysplasia of the hip: Early-term results from a prospective multicenter cohort. Journal of Pediatric Orthopedics.

[6] Schaeffer EK, Mulpuri K, IHDI Study Group (2018). Developmental dysplasia of the hip: addressing evidence gaps with a multicentre prospective international study. Medical Journal of Australia.

[7] Gibbard M, Zivkovic I, Jivraj B (2021). A global survey of patient and caregiver experiences throughout care for developmental dysplasia of the hip. Journal of Pediatric Orthopedics.

